# S15-2: Trends in Patterns of Participation in Club-Organized and Self-Organized Sports in Swedish Adolescents in 2013-2019

**DOI:** 10.1093/eurpub/ckae114.266

**Published:** 2024-09-26

**Authors:** Jennifer Gothilander, Lena Almqvist, Camilla Eriksson, Johanna Fritz

**Affiliations:** School of Health, Care and Social Welfare, Mälardalen University, Sweden; School of Health, Care and Social Welfare, Mälardalen University, Sweden; School of Health, Care and Social Welfare, Mälardalen University, Sweden; School of Health, Care and Social Welfare, Mälardalen University, Sweden

## Abstract

Adolescents’ participation in club-organized sports has decreased over time. Adolescents are known to drop out of club-organized sports as they age and may instead participate in self-organized sports. Boys are reported to participate more in club-organized sports than girls.

**Purpose:**

To study the trends of patterns of participation in club-organized and self-organized sports from 2013-2019 in Swedish adolescents aged 12-18 years.

**Methods:**

This study is part of a repeated cross-sectional study on sports and screen-time, including 3949 Swedish adolescents. Participation in club-organized and self-organized sports, watching TV & movies, and playing video games, was analyzed annually for the years 2013-2019, in 12–14-year-olds and 15-18-year-olds separately, by descriptive statistics, cluster analysis and, clusters’ centroids’ closeness across years. Clusters’ associations with gender were tested with regression analysis.

**Results:**

In younger adolescents, 68-78% participate in club-organized and 61-91% in self-organized sports. Among older adolescents, 52-64% participate in club-organized sports, and 70-88% in self-organized. In all years and both age groups, most adolescents are in patterns characterized by participating in sports. Patterns of inactivity and participation in sport are similar in 2013-2019. Younger adolescents show patterns of not participating in club-organized sports and older not in self-organized sports. The proportion of older adolescents in inactivity decreased over time. Among younger adolescents, no gender was frequently associated with any specific pattern, while girls were frequently associated with patterns of inactivity in the older age group.

**Conclusion:**

The study provides a new perspective on trends in patterns of participation in club-organized and self-organized sports from 2013 to 2019 in Swedish adolescents. Most adolescents were in patterns of participation in sports, yet the inactivity pattern was reoccurring. Younger adolescents showed patterns of not participating in club-organized sports and older not in self-organized sports. There were no gender differences in patterns among younger adolescents, while older girls are associated with patterns of inactivity. A policy that directly targets inactive adolescents, especially focusing on older girls, may be beneficial to increase participation in sports for all adolescents.

**Funding:**

Swedish Research Council (2018-05824_VR).

**Title 3:**

Growing grassroot participation opportunities in Kids’ Athletics through quality education and partnerships

**Purpose:**

Kids’ Athletics (KA), initiated in 2002, has engaged over 13 million children globally. It addresses the concerning statistic that 81% of youth and young people not meeting WHO's daily physical activity recommendations, to provide positive grassroots athletics experiences for mental and physical well-being.

**Project or Policy Description:**

Recognising the evolving needs of children and communities, the KA programme underwent a comprehensive review in 2021. The redesigned programme emphasises adaptability, inclusivity, enabling clubs, schools, sport organisations to foster participation through a modernised approach to athletics. The programme strategically utilises public spaces, eliminating the dependence on traditional athletics infrastructure and equipment, and employs a digital-first strategy for enhanced accessibility.

The KA approach is participant centred, providing positive experiences in physical activity to all children, no matter their individual abilities or circumstances. A positive experience is one that meets the individual needs of participants and is fun, safe, and teaches basic skills. Ensuring children and young people build the skills and confidence to by physically active for life.

Creating a capable and educated workforce was the priority in the initial implementation of the programme. In 2022/2023 World Athletics (WA) developed and conducted 'Train the Trainer' workshops across the globe, establishing an initial network of 200 KA National Leads in 157 countries, reaching over 500,000 children in 2023. Making training available online via the WA eLearning platform in late 2023 allowed WA to increase the reach of consistent information and training.

Kids’ Athletics is currently assessed based on programme reach, global participation, and accessibility for children. The next phase involves evaluating programme quality and impact across various contexts. As well as establishing local partnerships and collaboration with delivery partners and governments to scale up the delivery of the programme.

**Conclusion:**

Kids’ Athletics exemplifies grassroots sports delivery, offering quality physical activity opportunities through a physical literacy approach in clubs, schools, and communities.

